# Macrophage Depletion Lowered Blood Pressure and Attenuated Hypertensive Renal Injury and Fibrosis

**DOI:** 10.3389/fphys.2018.00473

**Published:** 2018-05-07

**Authors:** Lei Huang, Aimei Wang, Yun Hao, Weihong Li, Chang Liu, Zhihang Yang, Feng Zheng, Ming-Sheng Zhou

**Affiliations:** ^1^Department of Physiology, Shenyang Medical University, Shenyang, China; ^2^Department of Physiology, Jinzhou Medical University, Jinzhou, China; ^3^Department of Endocrinology, First Affiliated Hospital of Jinzhou Medical University, Jinzhou, China; ^4^Department of Nephrology, Second Affiliated Hospital of Dalian Medical University, Liaoning, China

**Keywords:** macrophage, proinflammatory cytokines, hypertension, renal injury, angiotensin II

## Abstract

Monocyte/macrophage recruitment is closely associated with the degree of hypertensive renal injury. We investigated the direct role of macrophages using liposome-encapsulated clodronate (LEC) to deplete monocytes/macrophages in hypertensive renal injury. C57BL/6 mice were treated with a pressor dose of angiotensin (Ang, 1.4 mg/kg/day) II plus LEC or the PBS-liposome for 2 weeks. Ang II mice developed hypertension, albuminuria, glomerulosclerosis, and renal fibrosis. LEC treatment reduced systolic blood pressure (SBP), albuminuria, and protected against renal structural injury in Ang II mice. Ang II significantly increased renal macrophage infiltration (MOMA2^+^ cells) and the expression of renal tumor necrosis factor α and interleukin β1, which were significantly reduced in Ang II/LEC mice. Ang II increased renal oxidative stress and the expression of profibrotic factors transforming growth factor (TGF) β1 and fibronectin. Ang II also inhibited the phosphorylation of endothelial nitric oxide synthase [phospho-endothelial nitric oxide synthesis (eNOS), ser1177]. LEC treatment reduced renal oxidative stress and TGFβ1 and fibronectin expressions, and increased phospho-eNOS expression in the Ang II mice. In Dahl rats of salt-sensitive hypertension, LEC treatment for 4 weeks significantly attenuated the elevation of SBP induced by high salt intake and protected against renal injury and fibrosis. Our results demonstrate that renal macrophages play a critical role in the development of hypertension and hypertensive renal injury and fibrosis; the underlying mechanisms may be involved in the reduction in macrophage-driven renal inflammation and restoration of the balance between renal oxidative stress and eNOS. Therefore, macrophages should be considered as a potential therapeutic target to reduce the adverse consequences of hypertensive renal diseases.

## Introduction

Hypertension is a major risk factor for nephrosclerosis and end-stage renal diseases (Appel et al., 2010). Despite extensive studies, the mechanisms by which hypertension causes renal injury are complicated and are not completely understood. Hypertensive nephropathy is characterized with renal inflammation, glomerular sclerosis, vascular hypertrophy, glomerular, and interstitial fibrosis (Yamashita et al., 2002; Liang et al., 2016). Recent studies suggest that immune cells, particularly for monocyte/macrophage lineage, play a critical role in the pathogenesis of hypertensive renal injury (Tian and Chen, 2015; Wenzel et al., 2016)

The mononuclear phagocyte system is the first line of immune cells in response to tissue injury, and plays an important role in tissue homeostasis and immune and non-immune-mediated tissue injury and repair (Mosser and Edwards, 2008; Mantovani et al., 2013). Infiltrated and activated monocytes/macrophages release chemokines and cytokines which may cause renal inflammation, endothelial dysfunction, glomerular and tubule sclerosis, and fibrosis (Wang and Harris, 2011). Macrophage accumulation is correlated with the degree of renal dysfunction and/or severity of renal fibrosis in several animal models of kidney diseases including glomerulonephritis, ischemia-reperfusion renal injury, and diabetic nephropathy (Wang and Harris, 2011; Zhang M.Z. et al., 2012; You et al., 2013; Huen and Cantley, 2017). Increased infiltration of monocytes/macrophages into the kidney has also been reported in various animal models of hypertension. Immunosuppressive therapies reduce blood pressure and the number of infiltrated macrophages in the kidney and improve renal function in a variety of experimental hypertensive animals (Tian et al., 2007; McMaster et al., 2015). Several studies using chemical and genetic modification of macrophages have demonstrated that elimination of macrophages improves endothelial function and reduces vascular oxidative stress and the deleterious cardiac and vascular remodeling in the hypertensive animals (Wenzel et al., 2011; Thang et al., 2015; Kain et al., 2016; Wenzel et al., 2016). However, there are still no studies that have manipulated the level of macrophages to examine their direct role in hypertensive renal damage.

Inappropriate activation of renin–angiotensin (Ang) system is implicated in the pathogenesis of hypertension and hypertensive kidney damage (Zhou et al., 2003; Schulman et al., 2006). Ang II facilitates the infiltration of monocytes/macrophages into the kidney and induces renal inflammation and injury (Rudemiller et al., 2016). Ang II increases renal oxidative stress, glomerular sclerosis, and renal fibrosis (Xia et al., 2014). It has been reported that reduced renal macrophage recruitment by the blockade or knockout of the CC chemokine receptor can attenuate Ang II-induced renal injury (Elmarakby et al., 2007; Liao et al., 2008). Liposome-encapsulated clodronate (LEC) is widely used for *in vivo* depletion of macrophages from various organs and tissues (Kitamoto et al., 2009). After being engulfed by macrophages, LEC accumulates in macrophages and induces macrophage apoptosis (Jordan et al., 2003). Here, we used chemical depletion of mononuclear phagocyte lineage by administrating LEC into two hypertensive animal models of Ang II mice and Dahl salt-sensitive (DS) rats to examine the direct role of macrophages in hypertensive renal injury. Our results support the idea that renal macrophages are main contributors to hypertensive renal injury and fibrosis.

## Materials and Methods

### Animal and Experimental Protocols

Six-week old male C57BL/6 mice or DS rats were purchased from Beijing Charles River Animal Laboratory (Beijing, China). All animal studies complied with the international standards stated in the Guide for the Care and Use of Laboratory Animals. All animal protocols were approved by the Institutional Animal Care and Use Committee of Jinzhou Medical University. The animals adapted to the new environment for 2 weeks before the experiments were performed.

### Animal Studies in Ang II Mice

The mice were randomly divided into four groups and treated for 2 weeks: (1) normotensive control (Ctr): sham surgery with implantation of an empty osmotic mini-pump plus an injection of PBS-liposome treatment (*n* = 8); (2) normotensive mice treated with LEC (LEC, Liposoma B.V., Amsterdam, Netherlands): implantation with an empty osmotic mini-pump plus LEC treatment (*n* = 8); (3) Ang II-infused mice (Ang II): implantation with an osmotic mini-pump of Ang II (1.4 mg/kg body weight/day, Sigma-Aldrich, St. Louis, MO, United States) plus PBS-liposome treatment (*n* = 8); (4) Ang II-infused mice treated with LEC (Ang II/LEC): implantation with an osmotic mini-pump of Ang II plus LEC treatment (*n* = 8). To implant the osmotic mini-pump (Alzet model 1002D, DURECT Inco., Cupertino, CA, United States), the mice were anesthetized using sodium pentobarbital (50 mg/kg I.P.). It has been reported that the mice receiving a sustained infusion of Ang II at this high dose can develop hypertension and renal injury (Liao et al., 2008; Zhang W. et al., 2012; Xiao et al., 2015). Macrophage depletions were performed by tail vein injections of LEC at the dose of 50 mg/kg body weight. LEC injection was done 1 day before mini-pump implantation and the injections were repeated every 3 days until the end of the experiments. The mice in the control group received a tail vein injection of PBS-liposome at similar interval and injection volume. At day 2 of LEC injection, blood was collected from tail vein for blood smears. The smear was stained with Giemsa (GS500; Sigma-Aldrich, St. Louis, MO, United States) and the amounts of monocytes, lymphocytes, and granulocytes were characterized according to their nuclear morphology, and counted by a blind observer. A total of 350 cells per smear were counted. Systolic blood pressure (SBP) was determined in the conscious mice using the tail cuff method (Softron Biotechnology Co., Ltd., Beijing, China) as described in our previous study (Zhou et al., 2015). Briefly, SBP was measured in a quiet and dark room. The mice were trained daily for 5 consecutive days prior to the implantation of the mini-pump. SBP was measured at three time points: at baseline (before Ang II administration or LEC injection), the fifth day, and the end of 2 weeks after the infusion of Ang II. At least five successive readings were recorded and averaged for each mouse. The day before the mice were euthanized, urine was collected by squeezing the animal bladder to stimulate urination, and the urine was collected on a metal plate. The ratio of urine albumin/creatinine was determined by albumin-to-creatinine ration assay kit following the manufacturer’s instructions (Shanghai Haoran Bio-Technology Co., Ltd., Shanghai, China). The mice were euthanized by overdose anesthetic agent (sodium pentobarbital 100 mg/kg I.P.).

### DS Rat Model of Salt-Sensitive Hypertension

DS rats were randomly divided into three groups and treated for 4 weeks as follows: (1) NS: the rats were fed a normal salt diet (0.5% NaCl, *n* = 8) plus PBS-liposome treatment; HS: the rats were fed a high salt (HS) diet (4% NaCl, *n* = 8) plus PBS-liposome treatment (*n* = 8); (3) HS/LEC: the rats were fed a HS diet plus LEC treatment. The macrophage deletions were performed by tail vein injections of LEC at the dose of 20 mg/kg body weight. LEC injections were started the day before the rats were given with HS treatment and repeated every 3 days until the end of the experiments. SBP was measured by tail cuff method as described above.

### Renal Histological Examination

The renal tissues were fixed in 4% paraformaldehyde in phosphate-buffered saline. The specimens were embedded in paraffin and cut into 4 μm thick section. Periodic acid-Schiff (PAS) staining was performed to evaluate the renal glomerular injury including glomerulosclerosis and mesangial matrix expansion (Zheng et al., 2004, 2006). The slides were photographed using an Olympus DS-41 microscope equipped with an Olympus DP-72 camera. A minimum of 20 images in one slide with at least one glomerulus per field were examined. A dark purple color in the glomeruli in each field was recognized as sclerosis. Percentage sclerotic area in glomeruli in each field was analyzed using the Image Probe Plus 6.0 image analysis system. The percentage area of sclerosis for 20 images in one kidney section was averaged as one single sample. Masson-Trichrome (Sigma-Aldrich, St. Louis, MO, United States) was carried out to evaluate renal fibrosis. Semi-quantitative analysis of the collagen contents in the renal tissues was assessed by evaluating percentage of positive stained areas with Image Pro Plus image analysis system. All tissue samples were evaluated independently by two reviewers who were not aware of the groups to which the animals belonged.

### Immunofluorescence Analysis

Renal **s**ections (4 μm) were cut from paraffin embedded tissues for immunofluorescence analysis. After deparaffinization and hydration, renal sections were microwaved for 30 min at 60°C for antigen retrieval. The sections were incubated with primary antibody against monocyte/macrophage marker 2 (1:100 dilution with TBST buffer, MOMA2, Abcam Inco.) overnight at 4°C, followed by incubation at 37°C for fluorescein (FITC)-conjugated goat anti-rat lgG (1:200 dilution with TBST buffer, Protein Tech.) for 1 h. MOMA2 is a marker of monocyte/macrophage in mice (Yun et al., 2004; Daniel et al., 2017). The nuclei were stained by counter-staining with DAPI. The section in negative control was only incubated FITC-conjugated lgG without primary antibody incubation. No fluorescence was detected in the negative control section. Monocytes/macrophages (MOMA2 positive cells) in renal tissues were viewed using a fluorescence microscope, and the MOMA2-positive cells were counted by two experienced reviewers who were blind to experimental groups. Twenty images in each section were examined and the number of positive cells per image was expressed by per mm^2^ area of the image.

### Nicotinamide Adenine Dinucleotide Phosphate (NADPH) Oxidase Assay

Nicotinamide Adenine Dinucleotide Phosphate oxidase activity in renal homogenates was determined by lucigenin-enhanced chemiluminescence (ECL) in the presence of NADPH substrate as previously described (Zhou et al., 2005). In brief, 20 μl of renal homogenates was added into 50 mmol/l phosphate buffer (PH 7.4) containing 1 mmol/l EGTA as an assay solution, the reaction was triggered by adding NADPH (100 μmol/l) substrate and lucigenin (5 μmol/L). The data were expressed as counts/mini/mg protein.

### Determination of *In Situ* **$O_{2}^{–}$** Production by Confocal Fluorescence Microscope

*In situ* $O_{2}^{–}$ production in renal tissues was determined by a confocal fluorescence microscope using oxidative fluorescent dihydroethidine (Sigma-Aldrich, St. Louis, MO, United States) as previously described (Zhou et al., 2003). In brief, the fresh renal tissues were embedded in OCT compound and cut into 5-μm-thick sections. The slides were submerged in 2 μmol/l dihydroethidine in HEPES buffer and incubated at 37°C for 30 min. The images were obtained with a Bio-Rad MRC-1024 laser scanning confocal microscope. A double-blind design was used to evaluate the image oxidative florescence intensity; the average fluorescent intensities were used for image quantification.

### Western Blot

Renal tissues were homogenized with lysis buffer containing 1 mmol/l PMSF, 10 μg/ml aprotinin, and 10 μg/ml leupeptin. After the homogenization, an aliquot of supernatant was taken for protein measurement with Bio-Rad protein assay. Thirty micrograms of protein was separated by SDS–PAGE and transferred to a nitrocellulose membrane. The membranes were incubated with blocking solution (5% milk added to TBST buffer) at room temperature for 1 h, then incubated with primary antibodies against phospho-endothelial nitric oxide synthesis (eNOS, Ser-1177, Cell signaling), NADPH oxidase subunits gp91phox and p22phox, tumor necrosis factor (TNF) α, interleukin (IL)1β, transforming growth factor (TGF) β1, and fibronectin (Santa Cruz Biotechnology Inc.) at 4°C overnight (all these primary antibodies were diluted by blocking solution in 1:500) and washed three times with TBST containing Tween 20, then the membranes were incubated with horseradish peroxidase-conjugated secondary antibody (1:2000 dilution using blocking solution) for 1 h at room temperature. The signals were detected by ECL using hyperfilm and ECL reagent (Santa Cruz Biotechnology Inc.). The membranes were reblotted for β-actin (1:500 dilution, Santa Cruz Biotechnology Inc.), to serve as a loading control. The data were normalized to β-actin and expressed as fold change versus control group.

### Statistical Analysis

The results were expressed as mean ± standard error of the mean (SEM). Statistical analyses were performed using SPSS 16.0 statistical software package (SPSS, Inc., Chicago, IL, United States), and statistical significance of difference was determined by two-way ANOVA with Bonferroni’s correction for multiple comparisons. Values were considered significant when *p* < 0.05.

## Results

### LCE Treatment Lowered Blood Pressure and Attenuated Ang II-Induced Albuminuria

**FIGURE 1:**
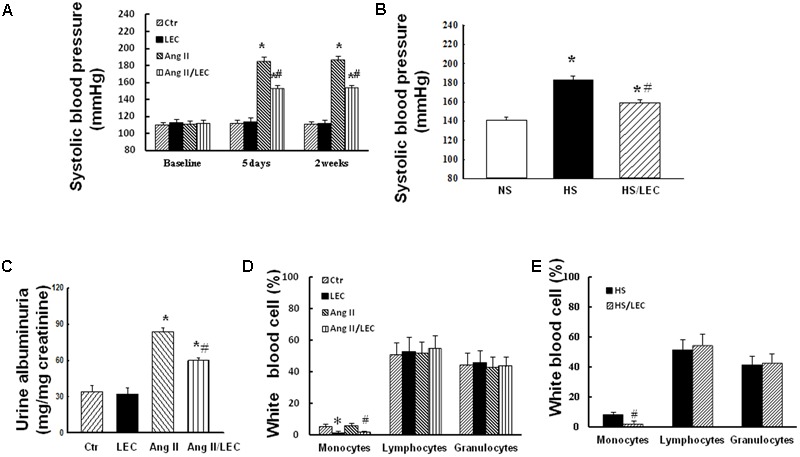
Treatment with liposome-encapsulated clodronate (LEC) reduced systolic blood pressure (SBP) in the angiotensin (Ang) II mice **(A)** and DS rats **(B)** and attenuated the ratio of albuminuria/creatinine **(C)** in the Ang II mice. Quantification of monocyte, lymphocyte, and granulocyte population in the circulating peripheral blood smear stained with Giemsa in Ang II mice **(D)** and hypertensive DS rats **(E)**. Ctr, control mice treated with PBS liposome; LEC, normal mice treated with LEC; Ang II, the mice treated with Ang II (1400 ng/min/mg body weight); Ang/LEC, the mice treated with Ang II plus LEC treatment; NS, DS rats fed a normal salt (0.5% NaCl) diet; HS, DS rats fed a HS (4% NaCl) diet. *N* = 8, ^∗^*p* < 0.05, vs. control group or NS group, ^#^*p* < 0.05, vs. Ang II group or HS group.

There was no significant difference in SBP among all groups of mice at baseline. SBP was significantly increased at the fifth day of Ang II infusion and maintained the high blood pressure for 2 weeks (187 ± 4 vs. 110 ± 3 mmHg in control, *p* < 0.05). LEC treatment significantly reduced SBP (154 ± 2 vs. 187 ± 4 mmHg in Ang II group, *p* < 0.05) in the Ang II-infused mice but not in control mice (**Figure 1A**). In DS rats, HS intake for 4 weeks significantly increased SBP (183 ± 4 vs. 141 ± 3 mmHg in NS, *p* < 0.05), LEC treatment significantly lowered SBP in HS rats (159 ± 3 vs. 183 ± 4 mmHg in HS, *p* < 0.05, **Figure 1B**). Infusion of Ang II for 2 weeks resulted in a significant increase in the ratio of albuminuria/creatinine (83.6 ± 2.3 vs. 33.9 ± 4.9 μg/mg creatinine in control group, *p* < 0.05). LEC treatment significantly reduced albuminuria in the Ang II infused mice but not in control mice (**Figure 1C**).

### LEC Effectively Reduced Circulating Monocytes and Renal Macrophages

**FIGURE 2:**
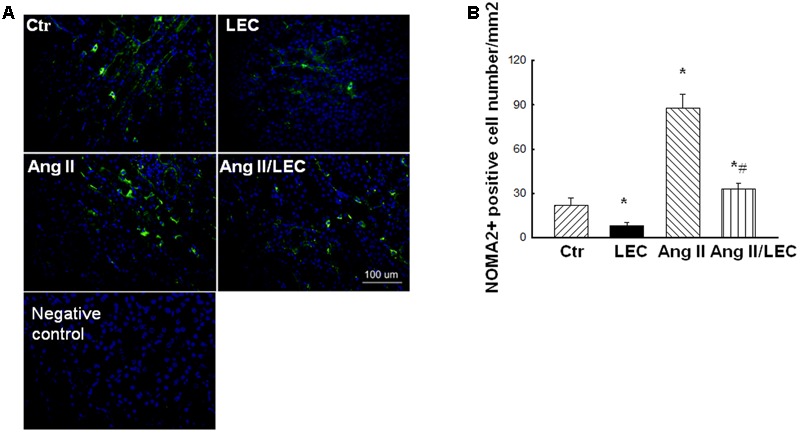
LEC treatment effectively reduced renal macrophages in Ang II mice. **(A)** Representative images of MOMA2 expression evaluated by immunofluorescence staining. **(B)** Quantitative assessment of MOMA2 expression in each group of mice. Green color indicates MOMA2 and blue color indicates DAPI. *N* = 8, ^∗^*p* < 0.05, vs. control group, ^#^*p* < 0.05 vs. Ang II group.

It has shown that LEC is an effective drug to deplete monocytes/macrophages via the induction of monocyte/macrophage apoptosis after it is engulfed by macrophages (van Rooijen and Hendrikx, 2010). In the present study, the mice were administrated with LEC at similar ways with the dose and the interval as reported (Jordan et al., 2003; Kitamoto et al., 2009; Moore et al., 2015; Kain et al., 2016). Consistent with these previous studies, the Giemsa-staining showed that LEC treatment reduced more than 70% circulating monocytes without significant changes in granulocyte and lymphocyte populations in the mice (**Figure 1D**). In DS hypertensive rats, LEC treatment also reduced the numbers of circulating monocytes by 67% without significant changes in other circulating cell populations (**Figure 1E**). Immunofluorescence showed that Ang II significantly increased the number of monocyte/macrophage marker MOMA2 positive cells in the renal tissues, LEC treatment significantly reduced the number of renal MOMA2 positive cells in both normotensive and Ang II-hypertensive mice (**Figure 2**), suggesting that LEC effectively and selectively targets monocyte/macrophage lineage.

### Depletion of Macrophages by LEC Protected Against Ang II-Induced Renal Injury and Fibrosis

**FIGURE 3:**
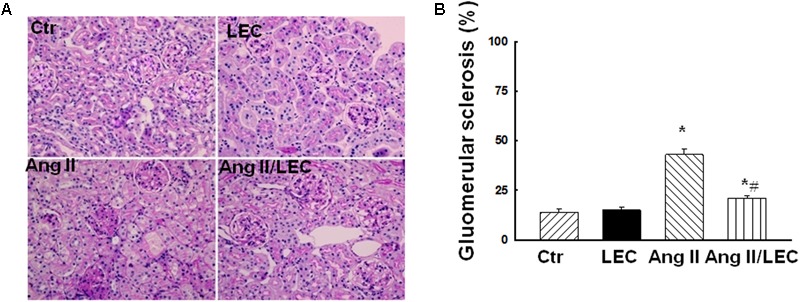
The macrophage depletion by liposome-encapsulated clodronate (LEC) attenuated Ang II-induced injury. **(A)** Representative images of kidney section stained with periodic acid-Schiff (PAS) for evaluation of glomerular injury. **(B)** Quantitative assessment of glomerular sclerosis in each group of mice. *N* = 8, ^∗^*p* < 0.05, vs. control group, ^#^*p* < 0.05, vs. Ang II group.

**FIGURE 4:**
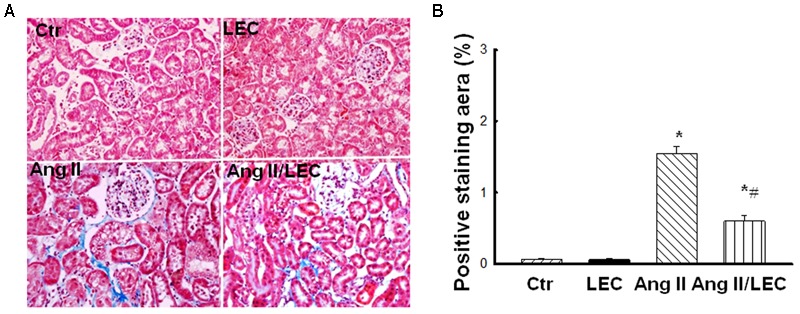
The macrophage depletion by LEC attenuated Ang II-induced renal fibrosis. **(A)** Representative the images of Masson-Trichrome-stained kidney sections for evaluation of renal fibrosis. **(B)** Quantitative assessment of renal fibrosis in each group of mice. *N* = 8, ^∗^*p* < 0.05, vs. control group, ^#^*p* < 0.05, vs. Ang II group.

To assess the effect of LEC treatment on Ang II-induced renal injury, renal tissues were stained with PAS and percentage area of glomerular sclerosis was calculated. As shown in **Figure 3**, compared with control mice, Ang II significantly increased glomerulosclerosis and glomerular matrix expansion, which was significantly attenuated by LEC treatment. Renal fibrosis is a hallmark of chronic hypertensive nephropathy. Inappropriate activation of RAS mainly by Ang II actions is considered as a major mechanism to contribute to renal fibrosis in hypertensive diseases (McMaster et al., 2015; Xiao et al., 2015). To determine the effect of macrophage depletion on renal fibrosis, Masson-Trichrome staining was performed. The quantification of collagen-stained area showed that Ang II infusion for 2 weeks significantly increased renal positive collagen-stained area and LEC treatment significantly attenuated the collagen-stained positive area (**Figure 4**). Although some investigators have reported that wild-type mice are to some extents resistance to Ang II-induced renal injury (Wesseling et al., 2005), an excess of Ang II can induce hypertension and profound renal damage (Zhang W. et al., 2012; Polichnowski et al., 2015; Xiao et al., 2015). The present study also supports that sustained infusion of Ang II at high dose can induce renal damage.

### LEC Treatment Attenuated Renal Injury and Fibrosis in Hypertensive DS Rats

**FIGURE 5:**
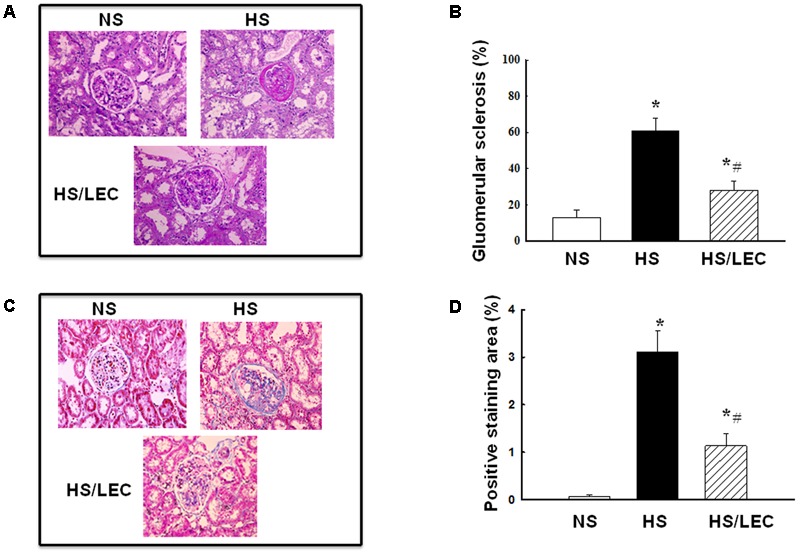
The macrophage depletion by LEC protected against renal injury and fibrosis in hypertensive DS rats. **(A)** Representative images of kidney section stained with periodic acid-Schiff (PAS) for evaluation of glomerular injury. **(B)** Quantitative assessment of glomerular sclerosis in each group of mice. **(C)** Representative images of kidney section stained with PAS for evaluation of glomerular injury. **(D)** Quantitative assessment of glomerular sclerosis in each group of mice. NS, DS rats were fed a normal salt diet; HS, DS rats were fed a HS diet; HS/LEC, DS rats were fed a HS diet with LEC treatment. *N* = 8, ^∗^*p* < 0.05, vs. NS group, ^#^*p* < 0.05, vs. HS group.

To confirm the renal protective effects of LEC, we used PAS staining and Masson-Trichrome staining to assess glomerular injury and renal fibrosis in hypertensive DS rats, respectively. As shown in **Figure 5**, hypertensive DS exhibited significant increase in glomerular sclerotic area with glomerular matrix expansion, which was significantly attenuated by LEC treatment. Renal fibrosis as demonstrated by collage-staining positive area was also increased in hypertensive DS rats, which reduced in HS/LEC rats (**Figures 5C,D**).

### LEC Effectively Reduced the Expression of Proinflammatory Cytokines TNFα and IL1β

**FIGURE 6:**
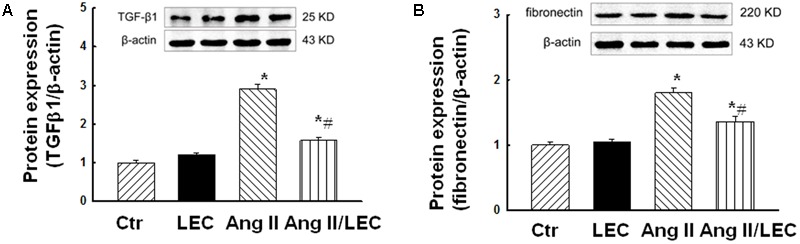
The macrophage depletion by LEC attenuated the protein expression of proinflammatory cytokines tumor necrosis factor (TNF) α **(A)** and interleukin β1 **(B)** in the kidney of the Ang II mice. *N* = 8, ^∗^*p* < 0.05, vs. control group, ^#^*p* < 0.05, vs. Ang II group.

It has been shown that macrophage-derived proinflammatory cytokines such as TNFα and IL1β play a crucial role in the induction of renal inflammation and injury (Vesey et al., 2002; Zhang et al., 2014). As shown in **Figure 6**, the protein expression of renal TNFα and IL1β was significantly increased in the Ang II mice, which was significantly reduced in the Ang II/LEC mice.

### LEC Reduced the Expressions of TGFβ1 and Fibronectin

**FIGURE 7:**
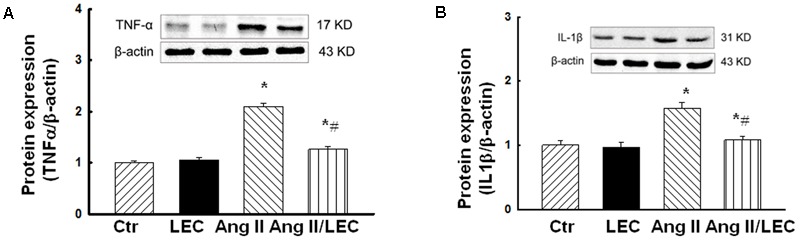
Macrophage depletion by LEC attenuated the protein expression of profibrotic factors tissue growth factor (TGF) β1 **(A)** and fibronectin **(B)** in the kidney of the Ang II mice. *N* = 8, ^∗^*p* < 0.05, vs. control group, ^#^*p* < 0.05, vs. Ang II group.

Transforming growth factor β1 and its downstream molecule fibronectin are important fibrotic factors which are considered as key mediators for renal fibrosis. As shown in **Figure 7**, Ang II infusion significantly increased the expressions of TGFβ1 and fibronectin, which were attenuated in the Ang II/LEC mice.

### Depletion of Macrophages by LEC Reduced Renal Oxidative Stress and Increased p-eNOS Expression in Ang II Hypertensive Mice

**FIGURE 8:**
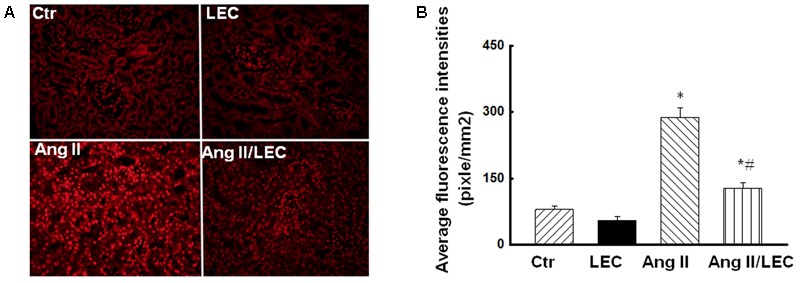
The macrophage depletion by LEC reduced oxidative fluorescence densities in the Ang II mice. **(A)** Representative images of renal sections stained with DHE for the evaluation of oxidative fluorescence density evaluated by confocal fluorescence microscope. **(B)** Quantification of average fluorescence densities in each group of mice. *N* = 8, ^∗^*p* < 0.05, vs. control group, ^#^*p* < 0.05, vs. Ang II group.

**FIGURE 9:**
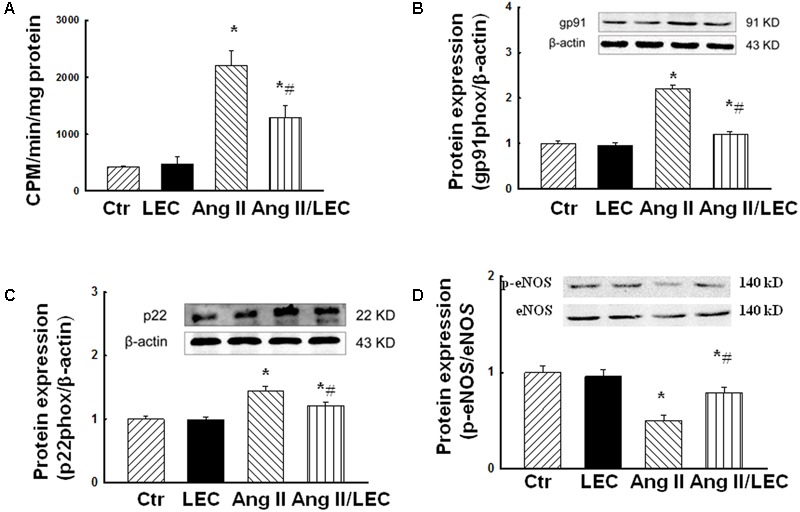
Effects of LEC treatment on NADPH oxidative activity **(A)**, the protein expression of renal NADPH oxidase subunits gp91phox **(B)** and p22phox **(C)**, and phospho-eNOS (Ser 1177, **D**) in Ang II mice. The macrophage depletion by LEC reduced renal NADPH oxidase activity and the expression of NADPH subunits gp91phox and p22phox, and preserved phospho-eNOS (Ser 1177) in the Ang II mice. *N* = 8, ^∗^*p* < 0.05, vs. control group, ^#^*p* < 0.05, vs. Ang II group.

The macrophages are major producers of reactive oxygen species (ROS) in the infiltrated tissues (Moore and MacKenzie, 2009; McMaster et al., 2015). Ang II increases ROS production via stimulation of NADPH oxidase (Rajagopalan et al., 1996). We assessed renal oxidative stress using confocal fluorescence microscope, NADPH assay, and the protein expression of NADPH oxidase subunits gp91phox and p22phox. As shown in **Figure 8**, renal oxidative fluorescence densities stained by dihydroethidine were significantly increased in the Ang II mice, and were significantly reduced in the Ang II/LEC mice. NADPH oxidase activity (**Figure 9A**) and the protein expressions of NADPH oxidase subunits gp91phox and p22phox (**Figures 9B,C**) were also significantly increased in the Ang II mice, and reduced in the Ang II mice treated with LEC. Phospho-eNOS (p-eNOS) at ser1177 is an active form of eNOS. The expression of p-eNOS and the ratio of p-eNOS/eNOS were significantly decreased in the Ang II hypertensive mice, which was also partly revised by LEC treatment (**Figure 9D**).

## Discussion

Hypertensive renal diseases are associated with the accumulation of macrophages in the kidney (You et al., 2013; Xiao et al., 2015). However, the direct role of macrophages in hypertensive renal diseases is not established. In the present study, we have demonstrated that: (1) the infusion of Ang II in the mice increased monocyte/macrophage recruitment in the kidney, LEC effectively and selectively targeted the circulating monocytes and reduced renal macrophages and inflammation; (2) the depletion of macrophages by LEC significantly reduced blood pressure and renal morphological injury and fibrosis in two hypertensive models of Ang II mice and DS rats; and (3) the depletion of macrophages attenuated Ang II-induced renal oxidative stress and preserved eNOS phosphorylation. These results support a direct role for macrophages in the pathogenesis of hypertensive renal damage.

Circulating monocytes and tissue macrophages play complex roles in the pathogenesis of hypertension (McMaster et al., 2015). In the vasculature and kidney, macrophage-derived ROS and inflammatory cytokines induce endothelial and epithelial dysfunction, respectively, resulting in vascular oxidative stress and impairment of sodium excretion, which may cause endothelial dysfunction and renal dysfunction, and contribute to hypertension (Wenzel et al., 2016). Using the mice lacking the macrophage colony stimulating factor (m-CSF), who are deficient in monocytes and macrophages, De Ciuceis et al. (2005) showed that these animals exhibited minimal elevation of blood pressure in response to chronic Ang II infusion and had preserved endothelium-dependent vasodilatation of the resistance mesenteric vessels. Ang II-induced hypertension is associated with increased inflammatory response and immune cell infiltration in the vasculature and the kidney (Rudemiller et al., 2016). Immunosuppressive therapy (Guan et al., 2013; Shah et al., 2015) or macrophage depletion (Moore et al., 2015) has been shown to rapidly inhibit Ang II-induced blood pressure elevation. The present study showed that the depletion of macrophages by LEC induced a rapid reversal of high blood pressure in Ang II mice. Our result demonstrates that the antihypertensive effects of LEC may be attributed in part to its inhibition of renal inflammation.

Macrophages have remarkable plasticity and heterogeneity (Mosser and Edwards, 2008). Macrophages mediate renal injury, mainly through classically activated (inflammatory) M1 macrophages (Mosser and Edwards, 2008; Mantovani et al., 2013). M1 macrophages are an important source of inflammatory cytokines in the infiltrated tissues (Wang et al., 2016; Wenzel et al., 2016). It has been shown that the mice lacking proinflammatory Th1 cells that produce interferon-γ (IFN-γ) and TNF-α were protected from hypertensive damage to the kidney glomerulus despite a preserved blood pressure response to Ang II (Zhang et al., 2014). TNFα is a pleiotropic cytokine which can control the production of other cytokines such as TGF1β in an autocrine and paracrine fashion (Awad et al., 2015). TNFα has been shown to play a critical role in diabetic and hypertensive renal injury (Singh et al., 2013; Awad et al., 2015). In the present study, Ang II increased the expression of the proinflammatory cytokines TNFα, IL1β (M1 marker), and fibrotic factor TGFβ1 (M2 marker). The depletion of macrophages by LEC reduced the expression of TNFα, IL1β, and TGF1β. Kitamoto et al. (2009) reported that LEC treatment reduced the expression of renal TNFα and TGFβ1, suggesting that LEC may not selectively target any specific macrophage subset. Recently, Moore et al. (2015) showed that Ang II hypertension in mice was associated with increased vascular M2 macrophage accumulation, which may differ with the findings in kidney that Ang II mainly increased proinflammatory M1 markers (Ruiz-Ortega et al., 2002; Mehaffey and Majid, 2017). Our results suggest that the reduction of renal injury and fibrosis after the depletion of macrophages may partially be achieved by the decrease in the macrophage-derived inflammatory cytokines and fibrotic factors.

Oxidative stress and inflammation are integrals of hypertension-induced renal injury (Awad et al., 2015; Ratliff et al., 2016; Pushpakumar et al., 2017). Infiltrated macrophages are major sources of ROS in the infiltrated tissues. Ang II increases oxidative stress by stimulating NADPH oxidase in the renal cells and/or the infiltrated macrophage (Keidar et al., 1995; Xu et al., 2016), NADPH oxidase in the phagocytes expresses large amount of gp91phox subunits (Moore and MacKenzie, 2009). Here we showed that the depletion of macrophages by LEC reduced Ang II-induced renal oxidative stress, inflammation, and renal injury in the Ang II mice. Consistent with our findings, a recent study (Pushpakumar et al., 2017) showed that the Ang II mice with Toll-like receptor (TLR) 4 deficiency, an important signaling molecule for activation of innate immune system, exhibited less renal injury and oxidative stress, suggesting an important link between renal inflammation, oxidative stress, and renal injury. Furthermore, we showed that the depletion of macrophages restored eNOS phosphorylation in the Ang II mice. We and others have demonstrated that the balance between eNOS-derived NO and oxidative stress is critical for the maintenance of cardiovascular and renal homeostasis (Patzak and Persson, 2007; Zhou et al., 2008). These results suggest that renal macrophages may promote renal injury by the induction of renal inflammation and the imbalance between renal oxidative stress and NO system.

### Limitations

The present study has several limitations. First, depletion of macrophages resulted in a significant reduction in SBP in two hypertensive animals with over 30 mmHg of maximal reduction in SBP. It is well known that hemodynamics are an important factor driving hypertensive renal injury (Polichnowski et al., 2015). Ang II-induced renal injury has BP-dependent and BP-independent components (Polichnowski and Cowley, 2009; Polichnowski et al., 2015) and in the present study, we did not control for BP changes in the LEC-treated mice. Therefore, the possibility that reduction in SBP *per se* may to some extents contribute to the amelioration of renal injury and dysfunction in hypertensive animals cannot be excluded. Next, we did not examine other immune cells or macrophage subset in the kidney; it has been reported that other immune cells such as T-lymphocytes may also play the role in the Ang II-induced hypertension and end organ damages (Barhoumi et al., 2011; Mian et al., 2016). However, because LEC selectively targets monocyte/macrophage lineage (Kain et al., 2016), the renal protection of LEC may mainly attribute to its depletion of macrophages other than the depletion of other immune cells. Third, we used lucigenin-ECL-based superoxide detection to measure NADPH oxidase activity. Although the method is frequently used by many other investigators, whether the method is specific for NADPH oxidase determination needs further clarification (Rezende et al., 2016). Finally, the present study was a prevention study but not an interventional study. Although the results from the present study support the notion that renal macrophages play an important role in hypertensive renal injury, treatment was initiated before the induction of disease and an interventional study may be required to provide evidence as to whether or not this approach can be used clinically to treat established disease.

### Future Directions

Although our studies provide evidence that depletion of monocytes/macrophages leads to obvious renal benefits, targeting all monocytes/macrophage would be of little therapeutic value because of the systemic immunosuppressive effects. Different macrophage subsets may play different role for renal diseases (Zhang M.Z. et al., 2012; Lech et al., 2014). Future study should identify the role of renal monocyte/macrophage subsets in hypertensive renal diseases, and refine techniques by using liposomes to target specific monocytes/macrophages for treatment of hypertensive or other renal diseases.

## Conclusion

The present study provides experimental evidence that renal macrophages are main mediators of renal injury and fibrosis and blood pressure elevation in two hypertensive animal models. Our data suggest that the underlying mechanisms may be macrophage production/release of cytokines and an imbalance between oxidative stress and NO in the kidney. While extrapolation between experimental animal studies and human hypertension is always speculative, since many of the animal models have been developed using the etiological factors which have been hypothesized to have a contributory role in human hypertension (Leong et al., 2015). Our studies support the notion that targeting renal macrophages (or macrophage subsets) might have important clinical and therapeutic implications for the treatment of hypertensive renal diseases.

## Author Contributions

LH contributed to the conception and design of the work; acquisition of data, analysis, and interpretation of data; statistical analysis. AW contributed to the acquisition of data, analysis, and interpretation of data and statistical analysis. YH, WL, CL, ZY, and FZ contributed to the acquisition of data, analysis, and interpretation of data. M-SZ contributed to the conception and design of the work, analysis, and interpretation of data and drafted the manuscript.

## Conflict of Interest Statement

The authors declare that the research was conducted in the absence of any commercial or financial relationships that could be construed as a potential conflict of interest.
